# Formation of tight junction-like structures of zonula occludens 2 in platelet–platelet interaction

**DOI:** 10.1016/j.rpth.2025.102845

**Published:** 2025-04-08

**Authors:** Magdolna Nagy, Markus Bender, Natalie S. Poulter, Jeremy A. Pike, Albert Sickmann, Sonja Vondenhoff, Natalia Bielicka, Marc A.M.J. van Zandvoort, Rory R. Koenen, Hugo ten Cate, Xavier Stéphenne, Johan W.M. Heemskerk, Constance C.F.M.J. Baaten

**Affiliations:** 1 Department of Biochemistry, Cardiovascular Research Institute Maastricht, Maastricht University, Maastricht, the Netherlands; 2 Institute of Experimental Biomedicine—Chair I, University Hospital Würzburg, Würzburg, Germany; 3 Department of Cardiovascular Sciences, School of Medical Sciences, College of Medicine and Health, University of Birmingham, Edgbaston, Birmingham, United Kingdom; 4 Centre of Membrane Proteins and Receptors (COMPARE), Universities of Birmingham and Nottingham, Midlands, United Kingdom; 5 The Research Software and Analytics Group, University of Exeter, Exeter, United Kingdom; 6 Department of Protein dynamics, Leibniz Institute for Analytical Sciences—ISAS-e.V, Dortmund, Germany; 7 Institute for Molecular Cardiovascular Research (IMCAR), University Hospital RWTH Aachen, Aachen, Germany; 8 Department of Biopharmacy and Radiopharmacy, Medical University of Bialystok, Bialystok, Poland; 9 Department of Molecular Cell Biology, Cardiovascular Research Institute Maastricht, Maastricht University, Maastricht, the Netherlands; 10 Department of Internal Medicine, Maastricht University Medical Centre+, Maastricht, the Netherlands; 11 Thrombosis Expertise Centre, Maastricht University Medical Centre+, Maastricht, the Netherlands; 12 Center for Thrombosis and Haemostasis (CTH), Gutenberg University Medical Center, Mainz, Germany; 13 Laboratory of Pediatric Hepatology and Cell Therapy, Institut de Recherche Expérimentale et Clinique (IREC), Université Catholique de Louvain, Brussels, Belgium; 14 Synapse Research Institute, Maastricht, the Netherlands

**Keywords:** actin cytoskeleton, integrin alpha IIb beta 3, platelets, tight junctions, zonula occludens 2

## Abstract

**Background:**

In tissues, cell–cell adhesion, barrier formation, and communication are regulated by gap, adherens, and tight junction (TJ) proteins. Platelets express several of these proteins. Platelets express key building blocks of gap junctions, the connexins, with known functions in integrin αIIbβ_3_ regulation. While, for some expressed TJ proteins like junctional adhesion molecule A and endothelial cell-specific adhesion molecule, a role in platelets has been uncovered, for other TJ proteins, like zonula occludens (ZO)-2 a contribution to platelet function is still unknown.

**Objectives:**

This study aimed to elucidate the role of ZO-2 in the stabilization of tight interplatelet contacts.

**Methods:**

Isolated human platelets from healthy volunteers and a patient deficient in ZO-2 were spread on fibrinogen and laminin surfaces in the presence of platelet agonists and inhibitors. Samples were fixed and prepared for microscopy.

**Results:**

Confocal and superresolution fluorescence microscopy indicated a redistribution of ZO-2 molecules forming clusters at sites of stable interplatelet contacts that was dependent on the platelet activation status. In the tight contacts, ZO-2 colocalized with endothelial cell-specific adhesion molecule and junctional adhesion molecule A. Furthermore, platinum replica electron microscopy revealed that interplatelet contacts resulted in compaction, detected as interwoven circumferential actin filaments, of interacting platelets. These changes were antagonized by cyclic adenosine monophosphate elevation and inhibition of αIIbβ_3_ integrins. In a blood sample from a patient deficient in ZO-2, we observed an increased thrombus stability, suggesting a potential regulation of thrombus stability by these TJ-like structures.

**Conclusion:**

Jointly, these data point to the assembly of TJ-like structures of interacting platelets, which enforce platelet adhesion contacts but lower 3-dimensional thrombus stability.

## Essentials

• Platelet–platelet interaction is mainly regulated by integrin αIIbβ_3_-fibrinogen binding.
• Platelets express proteins that are key parts of tight, adherens, and gap junctions in tissue.
• Tight junction proteins zonula occludens 2, endothelial cell-specific adhesion molecule, and junctional adhesion molecule A colocalize at platelet–platelet contact sites.
• *In vitro* thrombus formation experiments suggest a role of zonula occludens 2 in regulating thrombus stability.

## 1 Introduction

Blood platelets circulate as single entities that are prevented from interacting with each other. However, vascular damage results in dramatic changes in that platelets aggregate and form a thrombus via the initial establishment of interplatelet fibrinogen bridges [1,2]. *In vivo* and *in vitro,* the platelets in a thrombus show marked heterogeneity in responses and morphology [3,4]. Moreover, *in vivo* observations point to a separation of distinct regions within the thrombus, ie, a dense inner core and a looser outer shell [5]. In addition, the thrombus core consists of highly activated platelets that are tightly packed together, whereas the shell contains loosely adhered platelets of low activation stage [5]. How this structural heterogeneity is achieved is not fully understood.

For epithelial and endothelial tissues, the structural build-up is maintained by well-defined cell–cell interactions via 3 types of junctional complexes [6]. Adherens junctions and desmosomes anchor the cells, gap junctions (GJs) allow pore formation between adjacent cells to regulate intercellular communication, while tight junctions (TJs) or zonulae occludentes provide an intercellular sealing barrier, thereby regulating transcellular and paracellular transport processes. In well-studied tissue-forming cells, the adherens and TJ complexes appear to be formed in an interdependent way [7].

Similar to epithelial and endothelial cells, platelets are known to express key proteins that may function in GJ and TJ formation, such as connexins, junctional adhesion molecule (JAM)-A, and endothelial cell-specific adhesion molecule (ESAM) [[8], [9], [10], [11], [12], [13]]. Earlier mouse studies investigating the GJ proteins connexin-37 and connexin-40 pointed to a role of these in integrin αIIbβ_3_ activation, platelet aggregation, and thrombus formation. So far, it has remained unclear whether connexin-37 acts as a negative [10] or positive [8] regulator. Other mouse models showed that ESAM [12] and JAM-A [11,13], both proteins that in endothelial cells are implicated in TJ formation, negatively regulate platelet aggregation and limit thrombus growth. In particular, JAM-A was found to confine platelet activation processes by suppressing integrin αIIbβ_3_ activation [11]. Together, these observations raise the hypothesis that, also in platelets, GJ and TJ proteins are involved in the regulation of stable intercellular contacts. Early works by Skaer et al. [14] and Morgenstern [15] support this hypothesis as they revealed the presence of tight contacts in platelet aggregates formed upon ADP stimulation, where gap (3.0-5.0 nm) and tight contacts (0.0 nm) were identified along with focal contacts (40.0-50.0 nm: corresponding to the length of a fibrinogen molecule). Moreover, at the site of these tight contacts, Morgenstern observed the presence of the junction protein occludin [15].

Recent proteomics analyses revealed that platelets encompass an even wider range of junctional proteins [[16], [17], [18]]. Typical identified TJ proteins include zonula occludens (ZO)-2, claudin-3, and claudin-5, all of which are expressed in the order of 10^3^ copies per human platelet [18]. In tissue-forming cells, these proteins are known to make up the core of stable TJ, by linking junctional complexes to the actin cytoskeleton [19]. The TJ protein ZO-2 may have both structural and signaling functions, given the presence of 3 protein–protein interaction modules: 3 postsynaptic density-95, disc-large, ZO-1 (PDZ) domains, an SH3 domain, and a guanylate kinase domain [20]. How these proteins contribute to platelet–platelet interaction and thrombus formation is currently unknown.

In this study, we aimed to demonstrate the formation of TJ-like structures composed of ZO-2, ESAM, and JAM-A at sites of tight platelet–platelet contact. In spread platelets, these TJ-like structures elongate upon additional platelet activation, while platelet inhibition by cyclic adenosine monophosphate (cAMP) elevation or integrin inhibition disrupts the tight contacts. Using platelets from a patient deficient in ZO-2, we aimed to show that ZO-2 is necessary for the clustering of ESAM and JAM-A into TJ-like structures. Measurements of thrombus formation under flow pointed to a role of ZO-2 in the regulation of thrombus stability.

## 2 Methods

For further details, see Supplementary Material.

### 2.1 Blood collection and human platelet isolation

Citrate-anticoagulated blood was obtained from healthy volunteers and patients after informed consent in accordance with the tenets of the Declaration of Helsinki, according to protocols approved by the local medical ethics committees. Blood samples were obtained from a patient with progressive familial intrahepatic cholestasis (PFIC)4 with a homozygous mutation (c.1099C>T, p.[Arg367Ter], rs1057515613) in exon 7 of the *TJP2* gene (encoding ZO-2), resulting in a premature stop codon and, thereby, defective TJP2 expression. In addition, a blood sample was collected from a patient with PFIC2 caused by a homozygous missense mutation (c.2944G>A, p.[Gly982Arg], rs72549399) in exon 23 the *ABCB11* gene. From the patient with PFIC4, blood was collected before and after 6 weeks of treatment with odevixibat.

Platelet isolation was performed as previously described [21]. Platelet count was determined with a Sysmex XP300 hematology analyzer. For platelet phosphoproteome analysis of ZO-2, data were extracted from published databases [16,22].

### 2.2 Platelet spreading

Isolated platelets (15 × 10^6^/cm^2^ or 75 × 10^6^/cm^2^ for selected measurements) were spread on glass coverslips, precoated with fibrinogen (100 μg/mL) or laminin (100 μg/mL). In the case of inhibition of actin polymerization, isolated platelets were pretreated with latrunculin A (5 μM), cytochalasin D (10 μM), or vehicle control (dimethyl sulfoxide, 1:1000) for 5 minutes before platelets were added to the coated coverslips. After 5 minutes of initial adhesion, platelets were either left to spread in the presence of 2 mM calcium chloride (CaCl_2_) or additionally activated with 1 μM 2-methylthioadenosine diphosphate (2MeS-ADP), 15 μM thrombin receptor activator peptide-6 (TRAP-6), or 0.1 μg/mL crosslinked collagen–related peptide (CRP-XL) for 1 hour. Antagonists were either added before addition of platelet agonists for the duration of the experiment (tirofiban, 1 μg/mL) or added after 1 hour of spreading (iloprost, 1 μM; latrunculin A, 5 μM; cytochalasin D, 10 μM) or vehicle control (dimethyl sulfoxide, 1:1000) as appropriate) for 30 minutes.

### 2.3 Whole blood thrombus formation

Whole blood thrombus formation on collagen I at an arterial wall shear rate of 1000/s was performed using a previously described microfluidic assay [23]. The stability of thrombi on a collagen I surface was assessed by perfusing the preformed thrombi with HEPES buffer pH 7.45 (10 mM HEPES, 136 mM NaCl, 2.7 mM KCl, 2 mM MgCl_2_, 0.1% glucose, 0.1% bovine serum albumin [BSA]) containing 2 mM CaCl_2_ and 1 U/mL heparin for 10 minutes. Every 30 seconds, a bright field image was captured at the same spot using an EVOS FL microscope (ThermoFisher Scientific) equipped with a 60× oil objective (Olympus). Image analysis was with Fiji [24].

### 2.4 Immunofluorescence staining and confocal imaging

Spread platelets or cultured human coronary artery endothelial cells (HCAECs) were fixed with 1% paraformaldehyde, permeabilized with 0.005% sodium dodecyl sulfate, and subsequently blocked with 5% BSA in phosphate-buffered saline. The cells were then incubated overnight at 4 °C with combinations of the following antibodies in phosphate-buffered saline, containing 1% BSA: rabbit anti-ZO-2 polyclonal antibody (1:250), rabbit anti-claudin-5 monoclonal antibody (mAb) (1:200), mouse anti-ESAM mAb (1:250), mouse anti-JAM-A mAb (1:250), mouse anti-ZO-1 mAb (1:100), mouse anti-platelet and endothelial cell adhesion molecule-1 (PECAM-1) mAb (1:250), AF488-conjugated mouse anti-GPIbα Ab (1:250) or allophycocyanin-conjugated mouse anti-CD61 mAb (1:200). Incubation with secondary antibodies (2.5 μg/mL: AF488 goat anti-rabbit IgG and/or AF647 donkey anti-mouse IgG) and CF405M-conjugated phalloidin (1 unit/mL) was as appropriate, for 1 hour. Samples were embedded with glycerol mounting medium w/DABCO antifading agent. Confocal images were captured using a Leica DMI4000 equipped with a 63× oil immersion objective (NA 1.3) at an imaging speed of 400 Hz while averaging 3 images.

### 2.5 Image analysis

ZO-2 immunofluorescence images were assessed for the average length of ZO-2 clusters using the program Ilastik [25] and Fiji [24]. In short, using Ilastik, a random forest classifier was trained to separate the ZO-2 immunofluorescence images into 3 classes: background (no platelets), platelets (diffuse ZO-2 staining), and ZO-2 clusters based on randomly selected training images across the different conditions. Subsequently, the complete data set was analyzed with this classifier in Fiji, using a macro that determined the average length of the ZO-2 clusters. Fluorescence intensity profiles were assessed using Fiji [24].

### 2.6 Statistical analysis

Data are represented as median and IQR or mean ± SD. Graphpad Prism v10 was used for statistical analyses and visualization of data.

## 3 Results

### 3.1 Entanglement of circumferential actin filaments between contacting platelets

**Figure 1:**
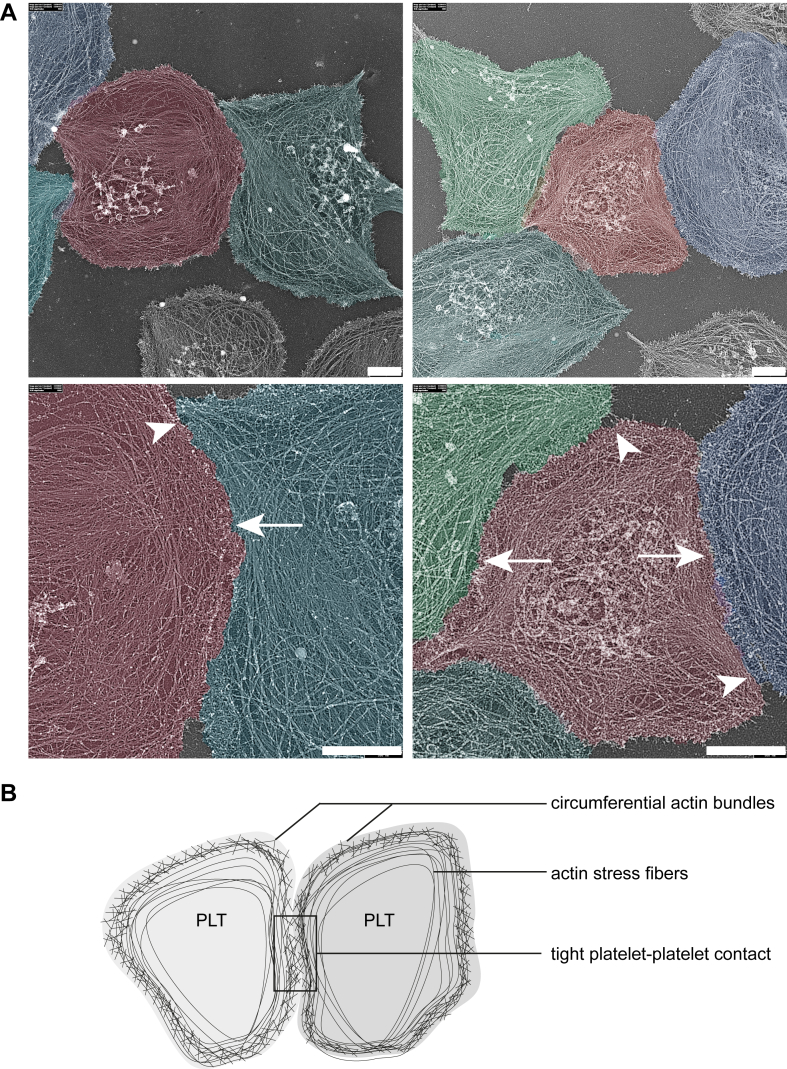
Upon platelet spreading, interacting platelets form tight contacts in which circumferential actin fibers interweave. (A) Spread platelets on fibrinogen were prepared for platinum replica (PR) electron microscopy (EM). Shown are representative PR-EM images (bar = 1 μm). For better visualization, pseudocoloring has been applied on original PR-EM images using Adobe Photoshop. Tight contacts and interwoven actin cytoskeletons are indicated by arrows, while gaps between interacting platelets are marked with arrowheads. (B) Diagram of tight contact formation between interacting platelets (PLTs) and actin cytoskeleton structures.

To investigate the tight contacts in platelet aggregates in further detail, we deployed an experimental setup that allowed spread platelets on a fibrinogen surface to interact. To specifically observe the reorganization of the actin membrane cytoskeleton upon interplatelet contact formation, we used platinum replica (PR) electron microscopy (EM) [26]. This method provides high-resolution information on cytoskeleton organization processes [27]. By applying PR-EM to samples of interacting human platelets, a clearly separated circumferential zone of F-actin was seen along the boundaries. Markedly, this actin zone appeared to be interwoven between platelets at sites of close intercellular contacts (Figure 1, arrows and diagram), suggesting that this zone shows interacting actin cytoskeleton elements between contacting platelets, ie, a process referred to as compaction [28].

### 3.2 Relocalization of cytosolic ZO-2 to sites of interplatelet contacts

**Figure 2:**
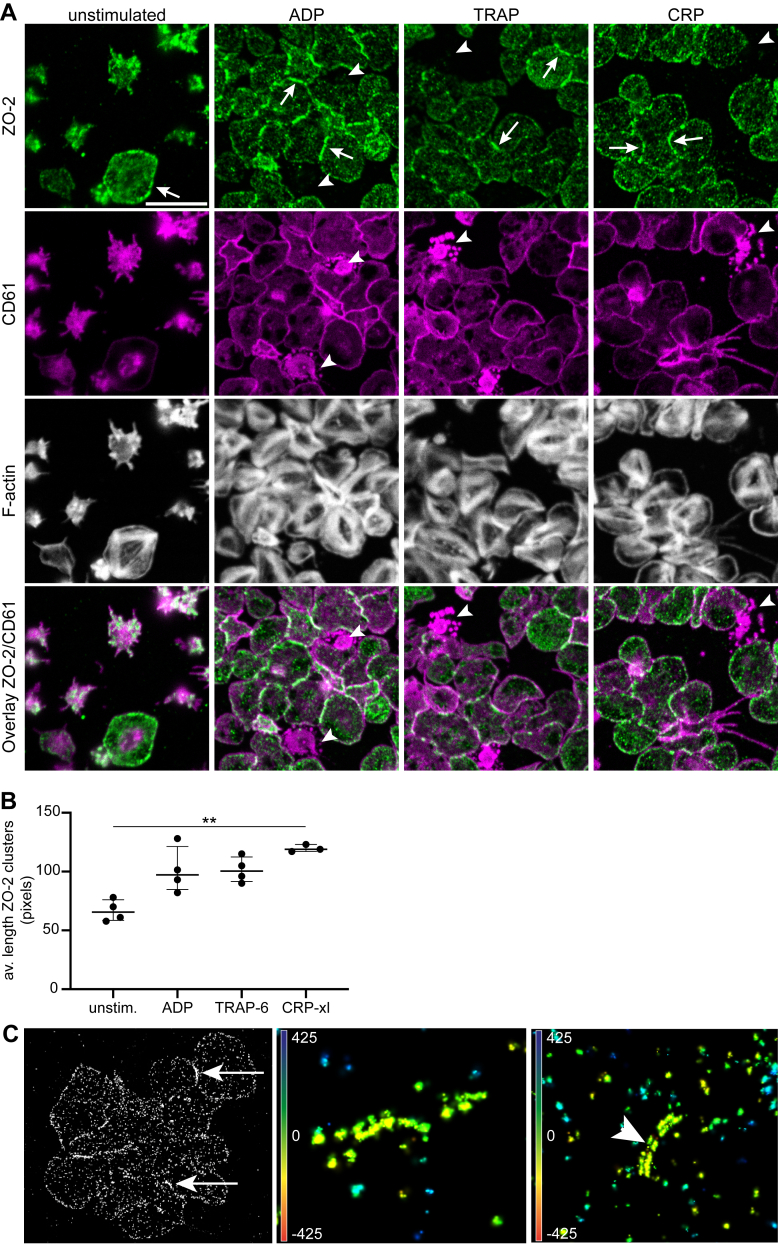
Relocalization of zonula occludens (ZO)-2 to the site of tight platelet–platelet contact upon spreading is sensitive toward the platelet activation status. (A) Relocalization of ZO-2 at contact sites between isolated platelets spread on fibrinogen (60 minutes) in the presence of 2 mM calcium chloride (CaCl_2_) with or without additional stimulation (1 μM 2-methylthioadenosine diphosphate [2MeS-ADP], 15 μM thrombin receptor activator peptide-6 [TRAP-6], or 0.1 μg/mL crosslinked collagen-related peptide [CRP-XL]). Platelets were fixed and immunostained for ZO-2 (green), CD61 (magenta), and F-actin (phalloidin, gray). Secondary antibody controls did not show staining (Supplementary Figure S6). Bar = 10 μm. Shown are representative images; full images are depicted in Supplementary Figure S1. Arrows indicate ZO-2 accumulation at sites of tight platelet–platelet contact; arrowheads point to microvesiculated platelets with absent ZO-2 staining. (B) Quantification of the average length of ZO clusters (in pixels) using Ilastik and Fiji. Data are illustrated as median plus IQR and analyzed using a Kruskal–Wallis test with a Dunn multiple comparisons test. ∗∗*P* < .01. *n*= 3–4 independent platelet donors; points indicate the average of at least 4 images per platelet donor per condition. (C) Platelets were allowed to spread on a fibrinogen surface as before. Subsequently, samples were prepared for 3-dimensional *d*STORM superresolution microscopy. Shown are molecular clusters of ZO-2 at interplatelet contact sites. Color code (yellow → blue) shows *z*-distance around fibrinogen surface.

Platelets express an abundant number of junctional proteins of which, mostly, a clear link to function has not been identified. To select proteins contributing to tight contacts of platelets, we made use of (phospho)proteome databases and literature. In platelets and megakaryocytes the *TJP2* gene is highly expresse [29]; it has an estimated protein abundance level of ∼4000 copies/platelet [18]. Furthermore, in platelets, targeted phosphoproteomics analyses identified multiple phosphorylation sites of the 160-kDa *TJP2* gene product ZO-2 that was regulated upon platelet inhibition and activation [16], suggesting regulation of ZO-2 in platelets. To obtain evidence for an intracellular localization of ZO-2 as TJ protein, human platelets were allowed to spread on fibrinogen, stained for ZO-2, and then visualized using confocal microscopy with a counterstain for F-actin (phalloidin) and CD61. In fully spread platelets without additional stimulation, we observed a few hotspots of ZO-2 staining at the platelet periphery (Figure 2A, arrows; Supplementary Figure S1). As phosphoproteomics analyses [16] showed differential phosphorylation patterns of ZO-2 upon stimulation with thrombin or thrombin+convulxin, spread platelets were additionally activated and stained for ZO-2. Supplementary Figure S2 shows an overview of ZO-2 phosphorylation sites regulated by platelet stimulation as published before [16,22,30]. Interestingly, when the platelets were stimulated with ADP, TRAP-6, or CRP-XL, resulting in a fully spread morphology, the formation of these ZO-2 clusters became more pronounced (Figure 2A, arrows; Supplementary Figure S1), and the average length of the ZO-2 clusters increased (Figure 2B). Additionally, we observed a loss in ZO-2 signal in those highly activated platelets that upon agonist stimulation had microvesiculated (Figure 2A, arrowheads; Supplementary Figure S1). Overall, the staining pattern of ZO-2 in activated, fully spreadout platelets resembled that of ZO-2 seen in a confluent layer of HCAECs (Supplementary Figure S3). Markedly, once platelets had interacted, the ZO-2 stain concentrated at the cell periphery, with highest accumulation at contact sites (Supplementary Figure S4). Moreover, in microaggregates formed on top of a layer of spread platelets in conditions with an increased platelet count ZO-2 clusters were visible between interacting, aggregating platelets (Supplementary Figure S5). Of note, no staining was present in negative controls using only secondary antibody (Supplementary Figure S6).

Superresolution 3-dimensional direct stochastic optical reconstruction microscopy (*d*STORM) was then used to acquire detailed molecular information on the local ZO-2 distribution of contacting platelets. This technique revealed strings of clustered ZO-2 molecules appearing at the areas of interplatelet contacts, which, in 3D perspective, were also close to the contact sites of platelets with underlying fibrinogen surface (Figure 2C, arrows). Interestingly, *d*STORM also showed that the ZO-2 enrichments consisted of 2 parallel strings of ZO-2 molecules (Figure 2C, arrowheads), suggesting relocalization of ZO-2 to the platelet periphery and subsequent alignment of patches of ZO-2 across the 2 platelets.

### 3.3 At sites of tight platelet–platelet contacts, ZO-2 colocalizes with ESAM and JAM-A

**Figure 3:**
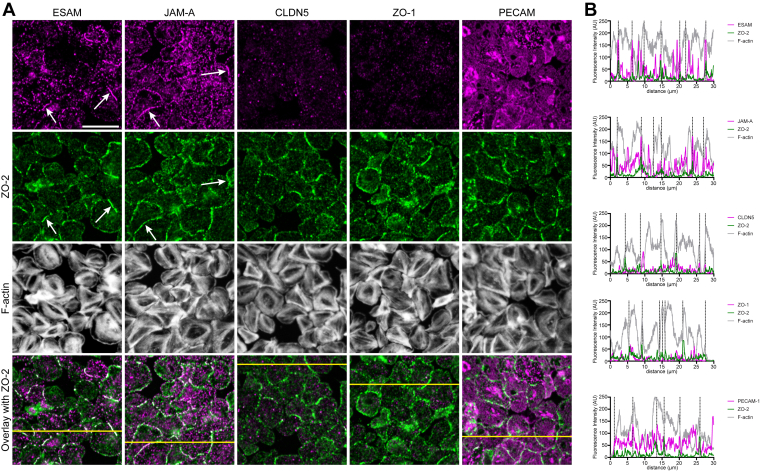
Colocalization of zonula occludens (ZO)-2 with endothelial cell-specific adhesion molecule (ESAM) and junctional adhesion molecule (JAM)-A in tight junction-like structures at sites of tight platelet–platelet contact. (A) Washed platelets (100 × 10^9^/L) spread on a fibrinogen surface in the presence of 2 mM calcium chloride (CaCl_2_) and 1 μM 2-methylthioadenosine diphosphate (2MeS-ADP), were fixed after 1 hour, and prepared for immunofluorescence staining. Samples were stained for ZO-2 localization (green) and F-actin (phalloidin, gray) in combination with an ESAM, JAM-A, claudin-5 (CLDN5), ZO-1, or platelet and endothelial cell adhesion molecule-1 (PECAM-1) antibody staining (magenta). Shown are representative images (full images are illustrated in Supplementary Figure S7). Scale bar is 10 μm. Arrows indicate colocalization of ZO-2 with ESAM and JAM-A. (B) Representative fluorescence intensity profiles taken along the yellow line shown in the overlays. Colors correspond to those in (A); vertical dotted lines denote the platelet boundaries.

In endothelial and epithelial tissue, TJs contain ZO-2 as an intracellular constituent along with multiprotein complexes spanning the plasma membrane [7]. To determine the composition of platelet TJ-like structures, ADP-activated platelets spread on a fibrinogen surface were stained for ZO-2 in combination with other known TJ (associated) proteins. HCAECs were included as a positive control (Supplementary Figure S3). In platelets, a high colocalization was observed for ZO-2 and ESAM as well as for ZO-2 and JAM-A (Figure 3A; Supplementary Figure S7, arrows). These observations were confirmed by fluorescence intensity profiles that showed colocalization of ESAM and JAM-A with ZO-2 at the platelet periphery at sites of tight platelet–platelet contacts (Figure 3B). In contrast, for claudin-5, ZO-1, and PECAM-1, we observed a different immunostaining pattern and no cluster formation at tight platelet–platelet contacts (Figure 3A; Supplementary Figure S7).

### 3.4 Platelet TJ-like contacts are dynamic structures regulated by actin polymerization and depolymerization

**Figure 4:**
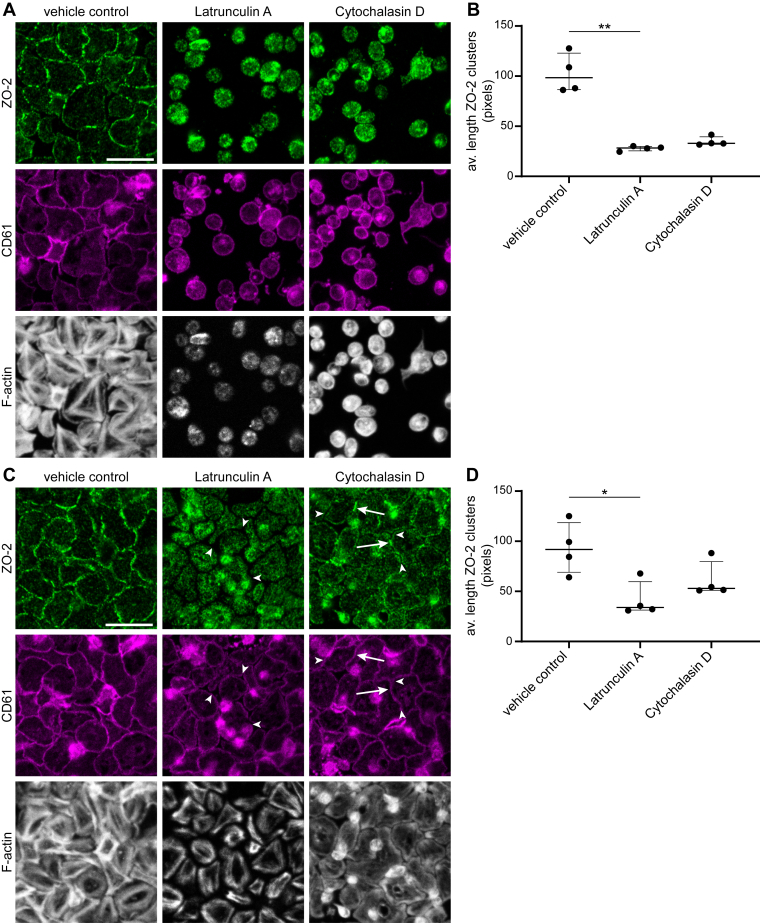
Zonula occludens (ZO)-2 clusters are dynamic structures that are controlled by polymerization and depolymerization of the actin cytoskeleton. Washed platelets were pretreated with latrunculin A or cytochalasin D and then allowed to adhere to a fibrinogen surface in the presence of 2 mM calcium chloride (CaCl_2_) and 1 μM 2-methylthioadenosine diphosphate (2MeS-ADP). Shown are representative confocal images (A) and corresponding quantification of average ZO-2 cluster length (B). Alternatively, ADP-stimulated spread platelets on a fibrinogen surface were posttreated with latrunculin A or cytochalasin D. Shown are representative confocal images (C) and corresponding quantification of average ZO-2 cluster length (D). ZO-2 staining is illustrated in green, while CD61 and F-actin (phalloidin) staining are depicted in magenta and gray, respectively. Shown are representative selections, with arrows pointing at ZO-2 clusters and tight contacts and arrowheads indicating gapping between adjacent platelets. Full images are illustrated in Supplementary Figure S8. Scale bar is 10 μm. Data are illustrated as median and IQR and analyzed using a Kruskal–Wallis test with a Dunn multiple comparisons test. ∗*P* < .05. *n*= 4 independent platelet donors; points indicate the average of at least 4 images per platelet donor per condition.

Disruptions in the actin cytoskeleton cause loosening of cell–cell TJs [19]. As merging of the circumferential actin filaments was observed between interacting platelets (Figure 1) and F-actin is known to directly interact with ZO-2 [31], the effect of disrupting the actin cytoskeleton on ZO-2 cluster formation was investigated in platelets. Actin polymerization was inhibited by addition of either latrunculin A or cytochalasin D. As expected, platelets treated with latrunculin A or cytochalasin D adhered to the fibrinogen surface but were unable to spread, although adjacent platelets still interacted. Interestingly, no ZO-2 clusters were visible at the periphery, and the average ZO-2 cluster length was significantly shorter with more pronounced effects of latrunculin A treatment (Figure 4A, B; Supplementary Figure S8A). Alternatively, platelets were allowed to spread and form contacts for 60 minutes, and then the actin cytoskeleton was disrupted by the addition of latrunculin A or cytochalasin D. In the case of latrunculin A posttreatment, adjacent platelets lost contact, as evidenced by the CD61 staining where gapping between platelets was observed (Figure 4C, white arrowheads; Supplementary Figure S8B). Remarkably, ZO-2 clusters at the platelet periphery had disintegrated and ZO-2 staining was observed more homogeneously throughout the platelets. Average ZO-2 cluster length reduced significantly upon latrunculin A posttreatment (Figure 4D). As for the cytochalasin D pretreatment, posttreatment with cytochalasin D also led to a less pronounced effect on the formation of tight platelet–platelet contacts. Although gaps formed between adjacent platelets (Figure 4C, white arrowheads; Supplementary Figure S8B), tight platelet–platelet contacts were still present after 30 minutes of cytochalasin D posttreatment (Figure 4C, white arrows; Supplementary Figure S8B). Concurrently, while ZO-2 cluster formation reduced (Figure 4D), some remaining ZO-2 clusters could still be observed at the site of platelet–platelet interactions (Figure 4C, white arrows; Supplementary Figure S8B). Whereas latrunculin A preferentially binds G-actin, cytochalasin D binds with high affinity to F-actin [32]. The observed differences between latrunculin A and cytochalasin D could possibly be due to their different mechanisms of action, resulting in different effects on the actin cytoskeleton (phalloidin staining, Figure 4C; Supplementary Figure S8B).

### 3.5 Reversible TJ formation between platelets by cAMP elevation

**Figure 5:**
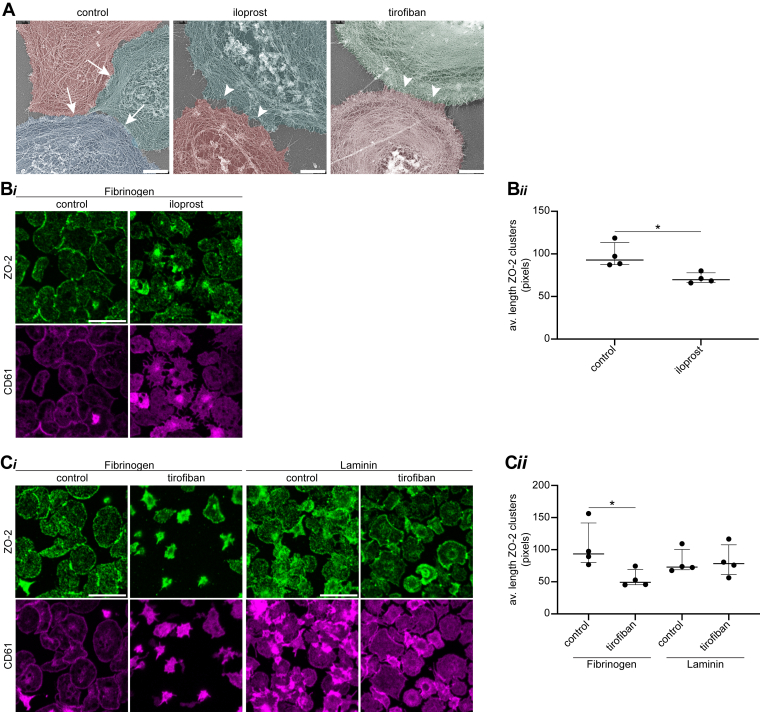
Disruption of zonula occludens (ZO)-2 clusters and tight platelet–platelet contacts by cyclic adenosine monophosphate (cAMP) elevation and integrin αIIbβ_3_ inhibition. (A) Platinum replica electron microscopy (PR-EM) demonstrating tight platelet–platelet contacts with interwoven circumferential actin zones (arrows) that are disrupted by iloprost or tirofiban treatment. Bar = 1 μm. For better visualization, pseudocoloring has been applied on original PR-EM images using Adobe Photoshop. Representative confocal images (Bi) and corresponding quantification of average ZO-2 cluster length (Bii) of adenosine diphosphate (ADP)-stimulated spread platelets on a fibrinogen surface posttreated with iloprost. Representative confocal images (Ci) and corresponding quantification of average ZO-2 cluster length (Cii) of ADP-stimulated spread platelets on a fibrinogen or laminin surface in the presence and absence of tirofiban. All samples (B and C) were stained for ZO-2 (green) and CD61 (magenta) localization. Shown are representative selections; full images are illustrated in Supplementary Figure S9. Scale bar is 10 μm. Data are illustrated as median and IQR and analyzed using a Mann–Whitney test (B) or a Kruskal–Wallis test with a Dunn multiple comparisons test (C). ∗*P* < .05. *n*= 4 independent platelet donors; points indicate the average of at least 4 images per platelet donor per condition.

For cultured endothelial monolayers, it has been described that cAMP elevation enhances the formation of TJ and diminishes paracellular permeability [33]. In phosphoproteomic analysis of iloprost-stimulated platelets, cAMP elevation and protein kinase (PK)A activation altered the phosphorylation of ZO-2 in the C-terminal and PDZ domains [22]. Markedly, of the 41 reported putative phosphosites in platelets (at serine, threonine, or tyrosine; UniProt-KB) [30], 22 of these are altered in response to the cAMP-elevating agent, iloprost. Four of these 22 contain a consensus PKA phosphorylation site, of which 2 (S174 and S244) are located in between the PDZ1 and PDZ2 domains, while the other 2 (S920 and S966) are near the C-terminus (Supplementary Figure S2) [22], suggesting also a role of cAMP in platelet TJ formation. To investigate this, spreading platelets were allowed to form contacts for 60 minutes, after which iloprost was administered. PR-EM and confocal microscopy indicated that this iloprost postaddition disrupted the tight platelet–platelet contacts (Figure 5A, Bi, arrowheads; Supplementary Figure S9A) and resulted in a significant shortening of the ZO-2 clusters (Figure 5Bii). The PR-EM images suggested that the cAMP elevation with iloprost caused secondary separation of the circumferential actin-rich zones between adjacent platelets (Figure 5A, arrowheads), hence pointing to a reversion of the compaction process.

### 3.5 Integrin αIIbβ_3_ activation is necessary for the formation of tight platelet–platelet contacts

Considering the central role of integrin αIIbβ_3_ in the regulation of platelet–platelet interactions, we next investigated its contribution to the formation of tight interplatelet contacts. To this end, platelets spreading on a fibrinogen or laminin surface were treated with tirofiban to block activation of integrin αIIbβ_3_. As expected, tirofiban strongly reduced the spreading on fibrinogen accompanied by a significant decrease in the number of ZO-2 clusters as well as the average length of the remaining clusters (Figure 5A, Ci-ii, arrowheads; Supplementary Figure S9B). In contrast, the relocalization of ZO-2 to the platelet periphery was not affected by tirofiban treatment when the platelets were spread on laminin (Figure 5Cii). Remarkably, fewer tight contacts between platelets had formed then (Figure 5B; Supplementary Figure S9B), suggesting that platelet–platelet interaction via integrin αIIbβ_3_ was a prerequisite for tight contact formation.

### 3.6 Platelet ZO-2 deficiency disturbs ESAM and JAM-A clustering at tight platelet–platelet contacts and increases thrombus stability

**Figure 6:**
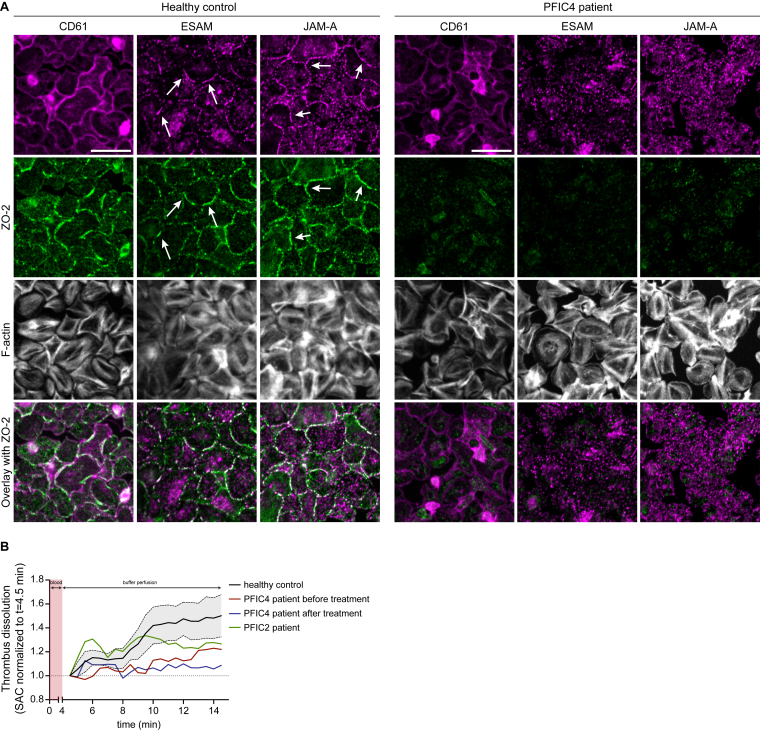
Endothelial cell-specific adhesion molecule (ESAM) and junctional adhesion molecule (JAM)-A cluster formation at sites of tight platelet–platelet contact is reduced in platelets from a patient with progressive familial intrahepatic cholestasis (PFIC)-4 and deficient in zonula occludens (ZO)-2, while thrombus stability is increased. (A) Washed platelets (100 × 10^9^/L) from a healthy donor and a patient with PFIC4 caused by a mutation in the *TJP2* gene coding for ZO-2 were spread on a fibrinogen surface in the presence of 2 mM calcium chloride (CaCl_2_) and 1 μM 2-methylthioadenosine diphosphate (2MeS-ADP). After 1 hour, platelets were fixed and stained for ZO-2 localization (green) and F-actin (phalloidin, gray) in combination with a CD61, ESAM, or JAM-A antibody staining (magenta). Full images are illustrated in Supplementary Figure S10. Scale bar is 10 μm. (B) Thrombi were formed on a collagen surface at shear rate of 1000/s using blood samples from healthy controls, a patient with PFIC4 before and after treatment with odevixibat, and a patient with PFIC2. Subsequently, the stability of the formed thrombi was determined over time. Shown is the dissolution of the formed thrombi during buffer perfusion in which thrombus dissolution is expressed as the surface area covered (SAC) by platelets and aggregates over time relative to platelet and aggregate SAC at *t* = 4.5 min. Data are mean ± SD for the healthy control group (*n* = 5).

To investigate the influence of ZO-2 deficiency on the formation of the TJ-like clusters at the site of tight platelet–platelet contacts, we obtained blood from a patient diagnosed with PFIC4 and defective ZO-2 expression, caused by a rare mutation in the *TJP2* gene. Platelet count and volume of the patient were within normal range (count 357 × 10^9^/L; mean platelet volume, 11.2 fL). The absence of ZO-2 was confirmed in platelets of the patients with PFIC4 (Figure 6A; Supplementary Figure S10). Upon platelet spreading on a fibrinogen surface in the presence of ADP, the defective platelets did not show the typical clustering of ESAM and JAM-A at the periphery, observed in the platelets from healthy volunteers (arrows). However, platelet spreading as such was not affected (Figure 6A; Supplementary Figure S10). We also explored the effects of platelet ZO-2 deficiency on thrombus dissolution and stability. The patient thrombi showed a greater stability over time (Figure 6B).

Patients with PFIC present with intrahepatic cholestasis and increased serum levels of bile acids [34]. As a control, we therefore obtained a blood sample from a patient with PFIC2 linked to an *ABCB11* gene mutation and another blood sample from the patient with PFIC4 after 6 weeks of treatment with odevixibat, an ileal bile acid transporter inhibitor that decreases bile acid reuptake in the intestines and thereby lowers serum bile acid levels [35].

Thrombus dissolution and stability of the ZO-2 deficient thrombi was not affected by odevixibat treatment (Figure 6B). Moreover, thrombus dissolution was greater for the patient with PFIC2 than for the patient with PFIC4 (Figure 6B), suggesting that the ZO-2 deficiency resulted in lesser thrombus dissolution and thus an increased thrombus stability.

## 4 Discussion

In this article, we present for the first time evidence of TJ-like structures at the sites of tight platelet–platelet contacts with an enriched expression of ZO-2, ESAM, and JAM-A. Our analysis of published phosphoproteomics data pointed to an extensive phosphorylation regulation of ZO-2 upon platelet activation as well as cAMP-dependent platelet inhibition. The multiple phosphorylation changes suggest that the ZO-2 protein is not merely a vestigial component inherited from the megakaryocyte but may contribute to key platelet functions. This was confirmed by our findings that, in a monolayer of interacting spread platelets, ZO-2 redistributes to the cell periphery, resulting in an enrichment of the protein at interplatelet contact sites that resembled that of TJs in confluent endothelial cells. This ZO-2 enrichment was sensitive to platelet activation as TJ-like clusters elongated upon additional platelet stimulation and disruption of the actin cytoskeleton by interfering in actin polymerization and depolymerization. Further, we found that elevation of cAMP resulted in a disintegration of platelet–platelet contacts, thus revealing an opposite regulation of TJ-like structures in platelets compared to endothelial tissue. Finally, using a blood sample from a patient with a rare deficiency in ZO-2, we observed a greater thrombus stability, suggesting a potential role of TJ-like structures in the regulation of thrombus stability.

In the late 1970s, Skaer et al. [14] investigated the intercellular space between platelets within an aggregate and distinguished 4 types of contact where the width of the intercellular space were 50.0, 20.0, 2.0 to 5.0, and 0.0 nm, respectively. They referred to the latter 2 types of contacts as gap and tight contacts [14]. Many years later, Vaiyapuri et al. [8,9] showed that connexins, ie, the building blocks of GJs, contribute to platelet aggregate formation and interplatelet communications [8,9]. To now further investigate the tight contacts, we used high-resolution techniques to analyze spreading and contacting of human platelets. Platelet spreading is characteristically subdivided in 4 stages: initial adhesion, formation of filopodia, formation of lamellipodia, and full spreading. In the third stage, a circumferential zone of orthogonally arrayed short actin filaments is known to be formed [36,37].

Interestingly, using high-resolution PR-EM, we observed a consistent merging of the actin circumferential zone at sites of immediate platelet–platelet contacts, reminiscent of the tight contacts identified by Skaer et al [14]. Upon treatment with iloprost, the zipper-like interwoven actin circumferential zones disentangled, resulting in a retraction of the membrane actin filaments in each cell. Crosscellular reorganization of the cytoskeleton is characteristic in the early stages of compaction, a process where the cells in a tissue become more compact [28]. In endothelium and epithelium, compaction and the initial formation of intercellular junctions are mediated by actin-driven lamellipodia [38,39]. Using superresolution *d*STORM microscopy, we observed for the first time similar processes in interacting platelets. In the process of lamellipodia-driven junction formation, important roles are reserved for the actin-linked proteins, ARP2/3, Rac1, WAVE, and Cdc42 [38,40,41]—all proteins that also play crucial roles in platelet lamellipodia formation [37,[42], [43], [44], [45]]. Moreover, F-actin and ZO-2 can directly associate [31]. In this study, we confirmed the important regulatory link between actin polymerization and ZO-2 cluster formation as interfering in actin (de)polymerization, after ZO-2 cluster formation had taken place, disrupted preexisting tight contacts and ZO-2 clusters at the periphery between interacting platelets.

For a long time, permanent platelet contacts were thought to be only regulated by integrin αIIbβ_3_–fibrinogen interactions. EM studies by Lewis et al. [46] in the early 1990s demonstrated an accumulation of integrin αIIbβ_3_ and fibrinogen in the vicinity of close platelet–platelet contacts [46]. We now show that also for the formation of tight contacts, integrin αIIbβ_3_ activation is necessary, as evidenced by the effects of tirofiban treatment on platelet–platelet interactions on a laminin surface. Our high-resolution and superresolution microscopy data extend these findings by showing that interacting platelets can also form TJ-like structures consisting of ZO-2, ESAM, and JAM-A, which resemble those present in confluent layers of endothelial cells. Remarkably, in spreading platelets from the patient with PFIC4 deficient in ZO-2, we observed a more diffuse staining pattern of ESAM and JAM-A, suggesting that platelet ZO-2 guides and/or keeps ESAM and JAM-A at the sites of contact, similar as was recently observed for ZO-2 and JAM-A in epithelial kidney cells [47]. Further, in the contacting platelets, claudin-5, PECAM-1, and ZO-1—present at TJs in other cell types—did not colocalize with ZO-2. Which other proteins beside ZO-2, ESAM, JAM-A, and occludin [15] are present at the TJ-like structures remains to be shown. Furthermore, like in other cells, platelet-expressed isoforms of ZO and connexin could interact and jointly make up the junction areas [[48], [49], [50]].

Our data show that the formation of the TJ-like structures is sensitive to the platelet activation status. Moreover, existing phosphoproteomic data show extensive phosphorylation of ZO-2 upon platelet inhibition and activation [16,22]. How phosphorylation affects ZO-2 activity and localization is currently not fully understood. However, it has been known for a long time that PKA activity regulates the TJ barrier function in different ways in a variety of cells [51,52]. Specifically for platelets, activation of PKA upon iloprost treatment results in phosphorylation changes in 22 amino acid residues [22], hence suggesting extensive regulation of this protein by the platelet-inhibiting PKA. In endothelium, cAMP elevation stimulates the formation of TJ and decreases paracellular permeability [33]. In contrast, we find that in platelets, cAMP elevation by iloprost resulted in decreased platelet–platelet contacts, separation of the circumferential actin filaments of adjacent platelets, and on average shorter ZO-2 clusters. For instance, endothelial cells—rather than platelets—possess an PKA-independent pathway of cAMP signaling, EPAC that upon activation, tightens intercellular junctions [53]. On the contrary, platelet activation with thrombin or thrombin/convulxin caused a markedly different change in phosphorylation pattern of ZO-2. In spread platelets that were additionally stimulated with ADP, TRAP-6, or CRP-XL, we observed an elongation of the ZO-2 clusters with the strongest effects obtained by CRP-XL stimulation. PKC is central in the regulation of TJ (dis)assembly and, depending on the isoform that is activated, either results in disassembly or consolidation of TJs [54]. Given the widespread role of the different PKC isoforms in platelet activation [55], PKC is thereby a plausible candidate for the regulation of the TJ-like structures in platelets, but further research is necessary. Intriguingly, in platelets that had microvesiculated upon additional stimulation, ZO-2 staining was absent. Whether this is connected to the increased ionophore-induced cleavage of ZO-2 detected in N-termini proteomics analysis [16] and altered phosphorylation pattern upon thrombin/convulxin stimulation remains to be shown.

Lastly, we studied *in vitro* thrombus formation in a patient with PFIC4 and observed that these patient thrombi deficient in ZO-2 were more stable over time. Additional control experiments with blood samples of the patient with PFIC4 upon odevixibat treatment to lower serum bile acid levels [35] and of a patient with PFIC2 caused by an *ABCB11* gene mutation, suggested that the deficiency in ZO-2 was responsible for the increased thrombus stability. Changes in the TJ-like structures could possibly alter the transport of platelet secretion products and plasma proteins throughout the thrombi, thereby influencing thrombus stability. Further, considering that both JAM-A and ESAM limit thrombus growth [11,12], changes in the TJ-like structures by a ZO-2 deficiency could potentially alter JAM-A-mediated and/or ESAM-mediated signaling.

Taken together, our data show a central role of the TJ protein ZO-2 in the formation and stabilization of tight contacts between adhered/spread platelets and contacting platelets in a thrombus, with potential roles of TJ-like structures in thrombus stability.
