# Supplementary material for: Formation of tight junction-like structures of zonula occludens 2 in platelet–platelet interaction
Source: Res Pract Thromb Haemost. 2025 Apr 8;9(3):102845. doi: 10.1016/j.rpth.2025.102845 (PMC12861806; doi:10.1016/j.rpth.2025.102845)
Supplement: Supplementary Material [file mmc1.pdf]

# Formation of tight junction-like structures of zonula occludens 2 in platelet-platelet interaction

Magdolna Nagy[1], Markus Bender[2], Natalie S. Poulter[3,4], Jeremy A. Pike[4,5], Albert Sickmann[6], Sonja Vondenhoff[7], Natalia Bielicka[8], Marc A. M. J. van Zandvoort[7,9], Rory R. Koenen[1], Hugo ten Cate[1,10,11,12], Xavier Stéphenne[13], Johan W. M. Heemskerk[1,14], Constance C. F. M. J. Baaten[1,7]

[1]Department of Biochemistry, Cardiovascular Research Institute Maastricht, Maastricht University, Maastricht, The Netherlands
[2]Institute of Experimental Biomedicine – Chair I, University Hospital Würzburg, Würzburg, Germany
[3]Department of Cardiovascular Sciences, School of Medical Sciences, College of Medicine and Health, University of Birmingham, Edgbaston, Birmingham, United Kingdom
[4]Centre of Membrane Proteins and Receptors (COMPARE), Universities of Birmingham and Nottingham, Midlands, United Kingdom
[5]The Research Software and Analytics Group, University of Exeter, Exeter, United Kingdom
[6]Department of Protein dynamics, Leibniz Institute for Analytical Sciences - ISAS-e.V, Dortmund, Germany
[7]Institute for Molecular Cardiovascular Research (IMCAR), University Hospital RWTH Aachen, Aachen, Germany
[8]Department of Biopharmacy and Radiopharmacy, Medical University of Bialystok, Bialystok, Poland
[9]Department of Molecular Cell Biology, Cardiovascular Research Institute Maastricht, Maastricht University, Maastricht, The Netherlands
[10]Department of Internal Medicine, Maastricht University Medical Centre+, Maastricht, The Netherlands
[11]Thrombosis Expertise Centre, Maastricht University Medical Centre+, Maastricht, The Netherlands
[12]Center for Thrombosis and Haemostasis (CTH), Gutenberg University Medical Center, Mainz, Germany
[13]Laboratoire d'Hépatologie Pédiatrique et Thérapie Cellulaire, Unité PEDI, Institut de Recherche Expérimentale et Clinique, Université catholique de Louvain (UCLouvain), Brussels, Belgium
[14]Synapse Research Institute, Maastricht, The Netherlands.

*Supplementary file*

## ***Supplementary materials and methods***

### *Materials*

Apyrase, calcium chloride, collagen G, bovine serum albumin (BSA), glucose, iloprost, human laminin, magnesium chloride, paraformaldehyde, SDS, human fibrinogen and dimethylsulfoxide were obtained from Merck (Darmstadt, Germany). Collagen type I came from Nycomed Pharma (Munich, Germany) and synthetic cross-linked collagen-related peptide (CRP-xl) was from Cambcol (University of Cambridge, Cambridge, United Kingdom). Thrombin Receptor Activating Peptide-6 (TRAP-6; SFLLRN) and 2MeS-ADP were from Tocris (Wiesbaden-Nordenstadt, Germany), while unfractionated heparin was from Leo Pharma (Ballerup, Denmark). Human coronary artery endothelial cells (HCAECs), endothelial cell growth medium MV2 and growth medium MV2 supplement mix were purchased from Promocell (Heidelberg, Germany). Cytochalasin D was from ThermoFisher (Waltham, MA, USA). Rabbit anti-ZO-2 antibody, rabbit anti-claudin-5 antibody, mouse anti-JAM-A antibody, mouse anti-ZO-1 antibody, mouse anti-PECAM-1 antibody, Alexa Fluor (AF)488 goat anti-rabbit IgG and AF647 donkey anti-mouse IgG were obtained from Invitrogen (Carlsbad, CA, USA). D-Phenylalanyl-L-prolyl-L-arginine chloromethyl ketone (PPACK) and latrunculin A was purchased at Abcam (Cambridge, United Kingdom) and the mouse anti-ESAM antibody at R&D systems (Minneapolis, MN, USA). Glycerol Mounting Medium w/DABCO and phalloidin conjugated with CF405-M were obtained from VWR (Radnor, PA, USA). AF488 mouse anti-GPIbα was from R&D Systems (Minneapolis, MN, USA).

### *Platelet isolation*

Platelet isolation was as described before.[1] In short, platelet-rich plasma was obtained by centrifuging citrate anticoagulated whole blood at 250 g for 15 min. PRP was supplemented with 1:10 v/v acidic citrate dextrose (ACD, 80 mM trisodium citrate, 52 mM citric acid and 180 mM glucose), and then centrifuged at 2230 g for 2 min, after which the platelet pellet was resuspended into Hepes buffer pH 6.6 (10 mM Hepes, 136 mM NaCl, 2.7 mM KCl, 2 mM

$MgCl_2$, 5 mM glucose and 0.1% BSA). After addition of 1:15 ACD and 0.1 U/mL (f.c.) apyrase, the platelets were centrifuged again at 2230 g for 2 min and subsequently resuspended into Hepes buffer pH 7.45 (10 mM Hepes, 136 mM NaCl, 2.7 mM KCl, 2 mM $MgCl_2$, 5 mM glucose and 0.1% BSA). Platelet count was determined with a Sysmex XP300 hematology analyzer (Kobe, Japan).

*Endothelial cell culture*

Human coronary artery endothelial cells (HCAECs: passage 5-6) were cultured under flow (10 dyn/$cm^2$) for three days in Ibidi µ-slides I Luer with a glass coverslip (#1.5H) and a channel height of 250 µm precoated with collagen G (40 µg/mL) to achieve endothelial alignment. Cells were cultured in endothelial cell growth medium MV2, supplemented with growth medium MV2 supplement mix at 37° C under 5% $CO_2$.

*Platinum replica electron microscopy (PR-EM)*

Platelets on coverslips were permeabilized for 5 min with PHEM buffer (60 mM Pipes, 25 mM Hepes, 10 mM EGTA and 2 mm $MgCl_2$) containing 0.75% Triton X-100, 1 µM phalloidin, 1 µM taxol and 0.1% glutaraldehyde, and finally incubated sequentially with 1% glutaraldehyde, 0.1% tannic acid, and 0.2% uranyl acetate. Samples were dehydrated by transferring through graded acetone. Critical point drying was performed in a Leica EM CPD300. Dried samples were covered under high vacuum with 1.2 nm platinum particles under rotation at 45°C, followed by 3 nm carbon particles at 90°C using a Leica EM ACE600 system. Platinum replicas were floated, picked up on formvar-carbon-coated grids, and examined with a JEOL JEM-2100 transmission electron microscope.[2] For better visualization, pseudo coloring was applied to the original grey PR-EM images using Adobe Photoshop.

*Super-resolution fluorescence microscopy*

For direct stochastic optical reconstruction microscopy (*d*STORM), stained platelet samples (see paragraphs ‘Platelet spreading’ and ‘Immunofluorescence staining’) were incubated with

PBS, supplemented with 2-mercaptoethylamine HCl (100 mM), glucose oxidase (50 µg/mL) and catalase (1 µg/mL) to induce fluorophore blinking. Single-molecule images of ZO-2 were acquired using a Nikon STORM system and 100x objective, in 3D *d*STORM mode. Reactivation of fluorophore blinking occurred by increasing laser power (405 nm) by 5% every 30 s, as described before.[3] The *d*STORM images were reconstructed by a Nikon STORM analysis module v3.2, using drift correction and Gaussian rendering.

### *References*


1 Baaten CCFMJ, Swieringa F, Misztal T, Mastenbroek TG, Feijge MAH, Bock PE, Donners M, Collins PW, Li R, van der Meijden PEJ, Heemskerk JWM. Platelet heterogeneity in activation-induced glycoprotein shedding: functional effects. *Blood Adv*. 2018; **2**: 2320-31. 10.1182/bloodadvances.2017011544.

2 Spindler M, van Eeuwijk JMM, Schurr Y, Nurden P, Nieswandt B, Stegner D, Reinhold A, Bender M. ADAP deficiency impairs megakaryocyte polarization with ectopic proplatelet release and causes microthrombocytopenia. *Blood*. 2018; **132**: 635-46. 10.1182/blood-2018-01-829259.

3 Poulter NS, Pollitt AY, Davies A, Malinova D, Nash GB, Hannon MJ, Pikramenou Z, Rappoport JZ, Hartwig JH, Owen DM, Thrasher AJ, Watson SP, Thomas SG. Platelet actin nodules are podosome-like structures dependent on Wiskott-Aldrich syndrome protein and ARP2/3 complex. *Nat Commun*. 2015; **6**: 7254. 10.1038/ncomms8254.

4 UniProt. Q9UDY2 ZO2_HUMAN.

5 UniProt Consortium. UniProt: the Universal Protein Knowledgebase in 2025. *Nucleic Acids Res*. 2025; **53**: D609-d17. 10.1093/nar/gkae1010.

6 Solari F, Mattheij NJ, Burkhart JM, Swieringa F, Collins PW, Cosemans JM, Sickmann A, Heemskerk JW, Zahedi RP. Combined quantification of the global proteome, phosphoproteome, and proteolytic cleavage to characterize altered platelet functions in the human scott syndrome. *Mol Cell Proteomics*. 2016; **15**: 3154-69.

7 Swieringa F, Solari FA, Pagel O, Beck F, Huang J, Feijge MAH, Jurk K, Körver-Keularts I, Mattheij NJA, Faber J, Pohlenz J, Russo A, Stumpel C, Schrander DE, Zieger B, van der Meijden PEJ, Zahedi RP, Sickmann A, Heemskerk JWM. Impaired iloprost-induced platelet inhibition and phosphoproteome changes in patients with confirmed pseudohypoparathyroidism type Ia, linked to genetic mutations in GNAS. *Sci Rep*. 2020; **10**: 11389. 10.1038/s41598-020-68379-3.

## *Supplementary Figures*

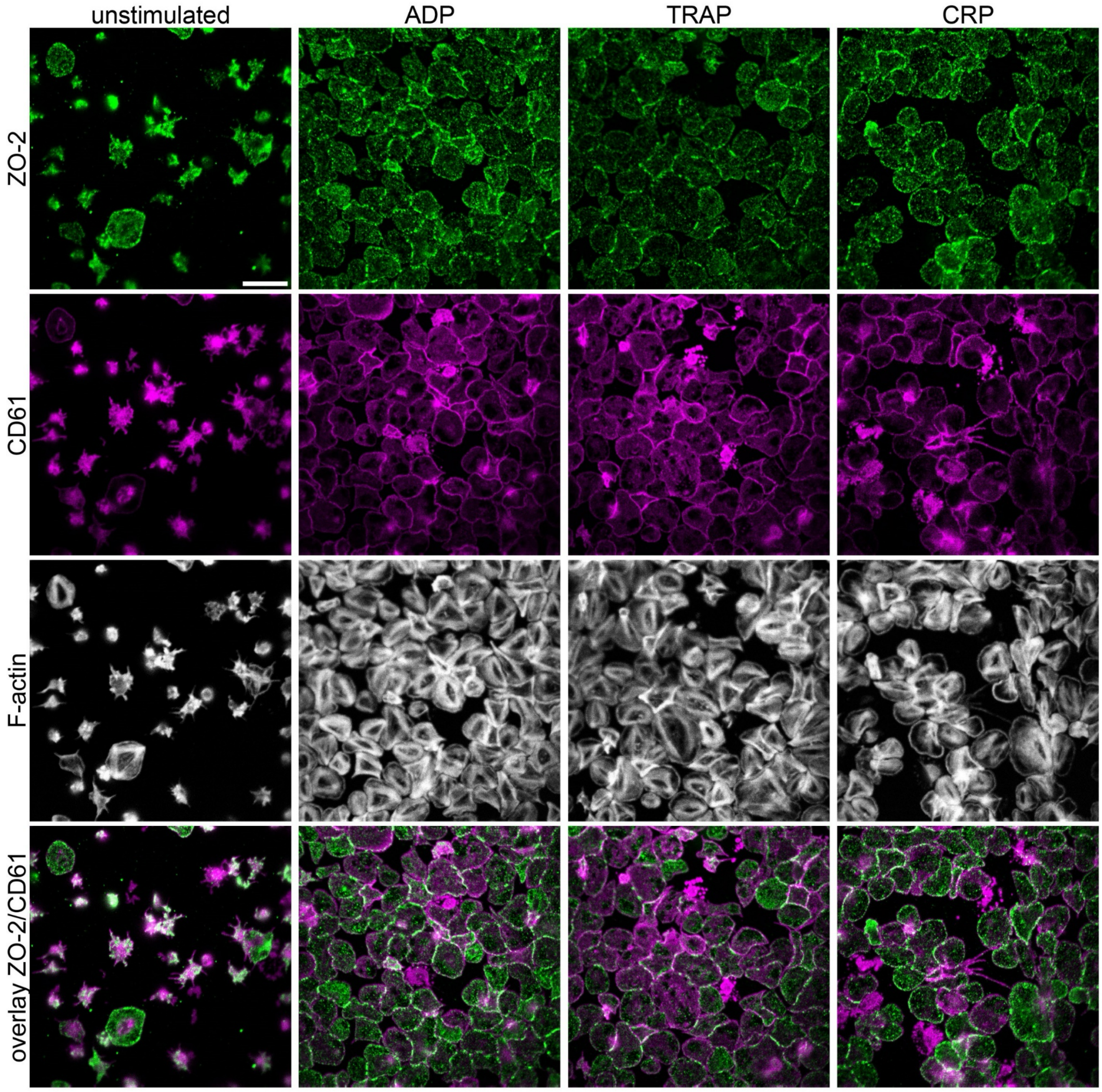


**Supplementary Figure S1. ZO-2 cluster formation at the site of platelet-platelet contact.** Washed platelets (100 x $10^9$/L) were allowed to spread on a fibrinogen surface in the presence of 2 mM $CaCl_2$ (unstimulated) with or without additional stimulation by 1 µM 2MeS-ADP, 15 µM TRAP-6 or 0.1 µg/mL CRP-xl. After one hour, platelets were fixed and stained for ZO-2 (green), CD61 (magenta) and F-actin (phalloidin, gray). Shown are representative images in full (magnifications of selected areas and quantification of ZO-2 clusters are illustrated in Figure 2). Scale bar is 10 µm.

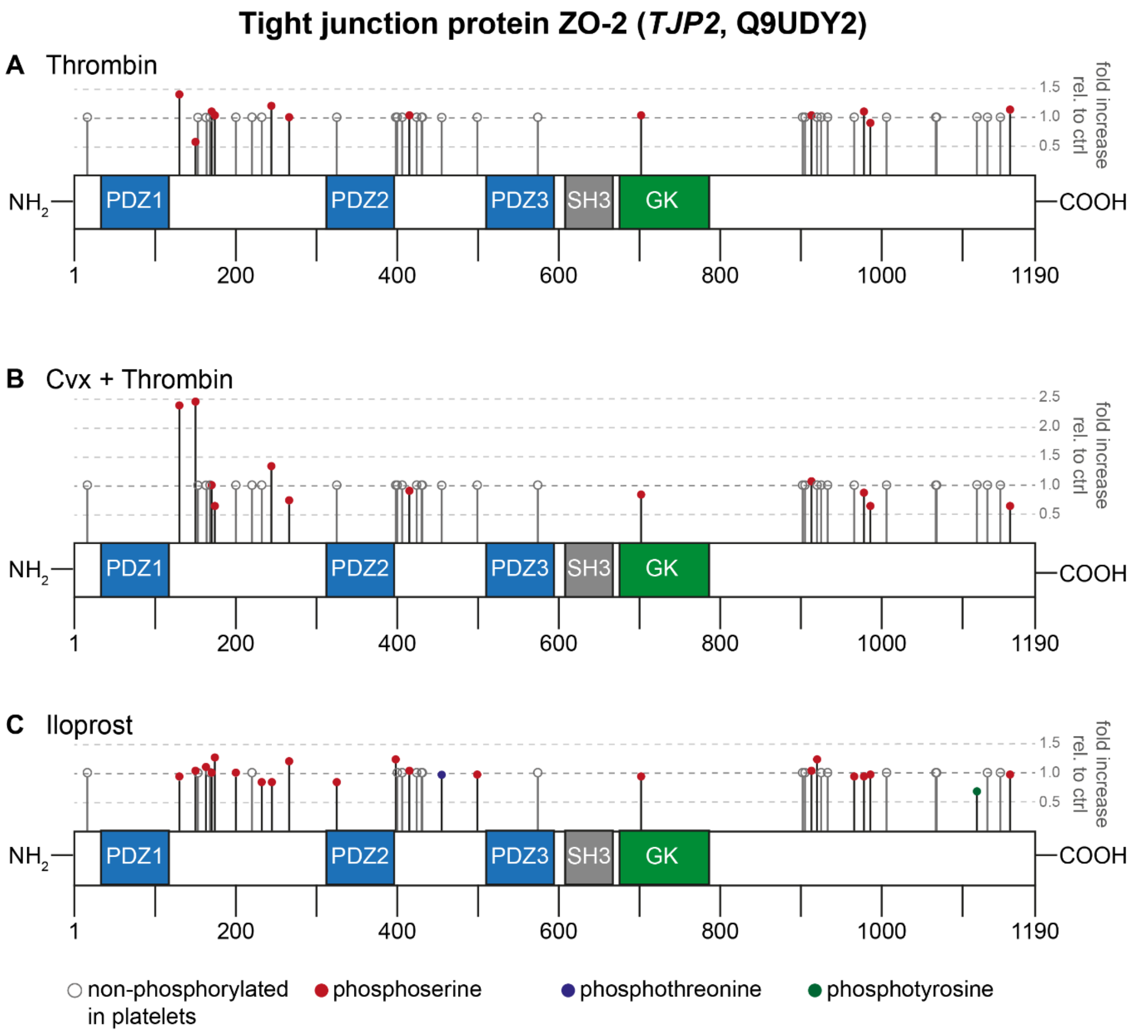


**Supplementary Figure S2. Altered phosphorylation profiles of ZO-2 identified in phosphoproteome indicate differential regulation of ZO-2 upon platelet inhibition and activation.** Schematic representation of the ZO-2 protein with PDZ and guanylate cyclase (GK) domains. All phosphosites of ZO-2 as described in UniProt are plotted.[4, 5] Phosphosites that are not regulated in platelets are indicated in grey, while serine, threonine and tyrosine residues that are phosphorylated upon platelet signaling are indicated in red, blue and green respectively. Fold changes in phospho-signal upon **A**) thrombin, **B**) thrombin/convulxin or **C**) iloprost treatment are indicated relative to that in untreated platelets (control) according to published data.[6, 7]

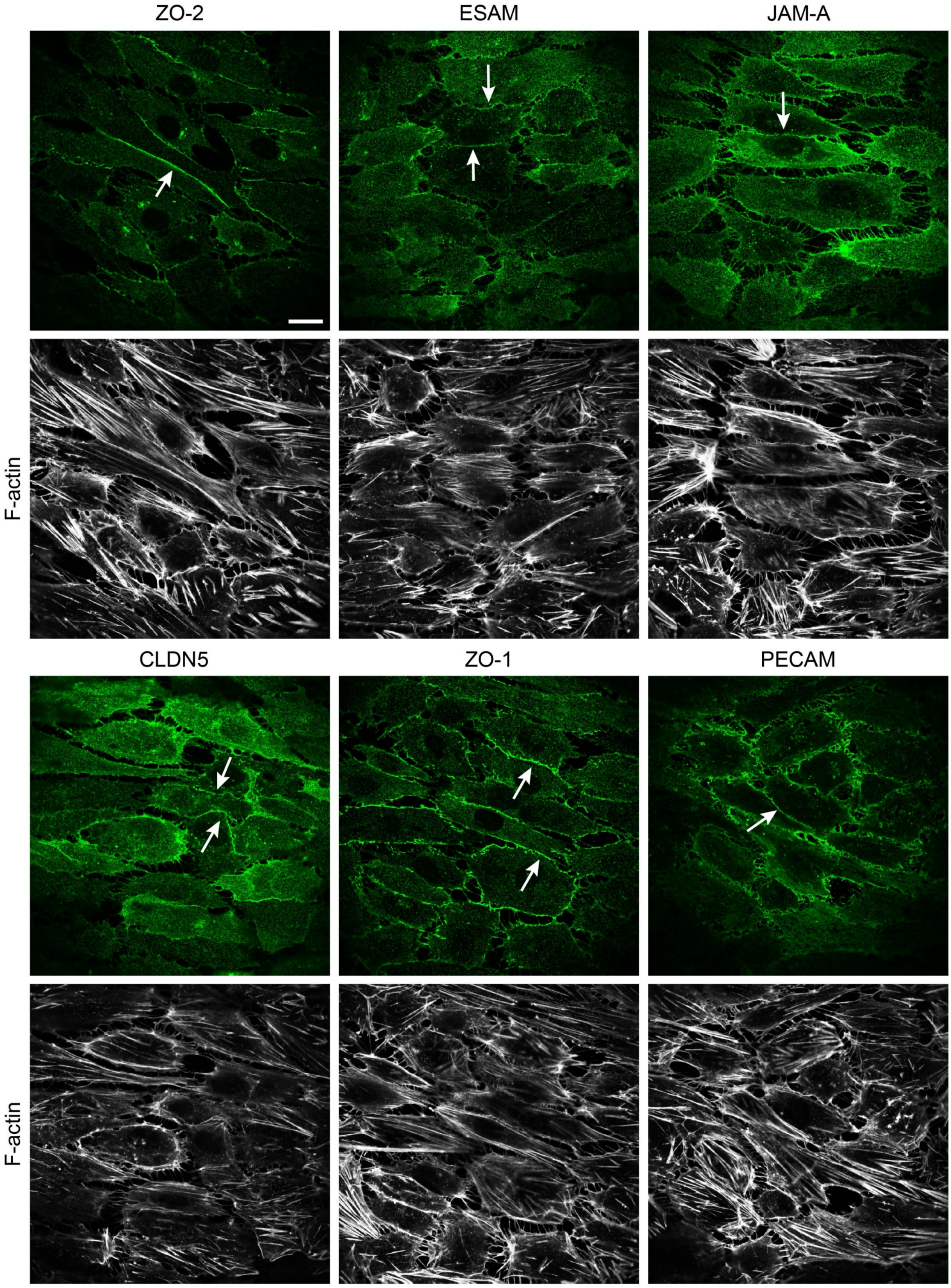


**Supplementary Figure S3. Human coronary artery endothelial cells as positive control for TJ staining.** Human coronary artery endothelial cells (HCAECs) were cultured under flow on a collagen G surface until confluent. HCAECs were fixed and stained as positive control for ZO-2, ESAM, JAM-A, claudin-5 (CLDN5), ZO-1 and PECAM-1 (green). Phalloidin was used to

counterstain the actin cytoskeleton (gray). Shown are representative confocal images with white arrows pointing towards tight junctions, scale bar = 20 µm.

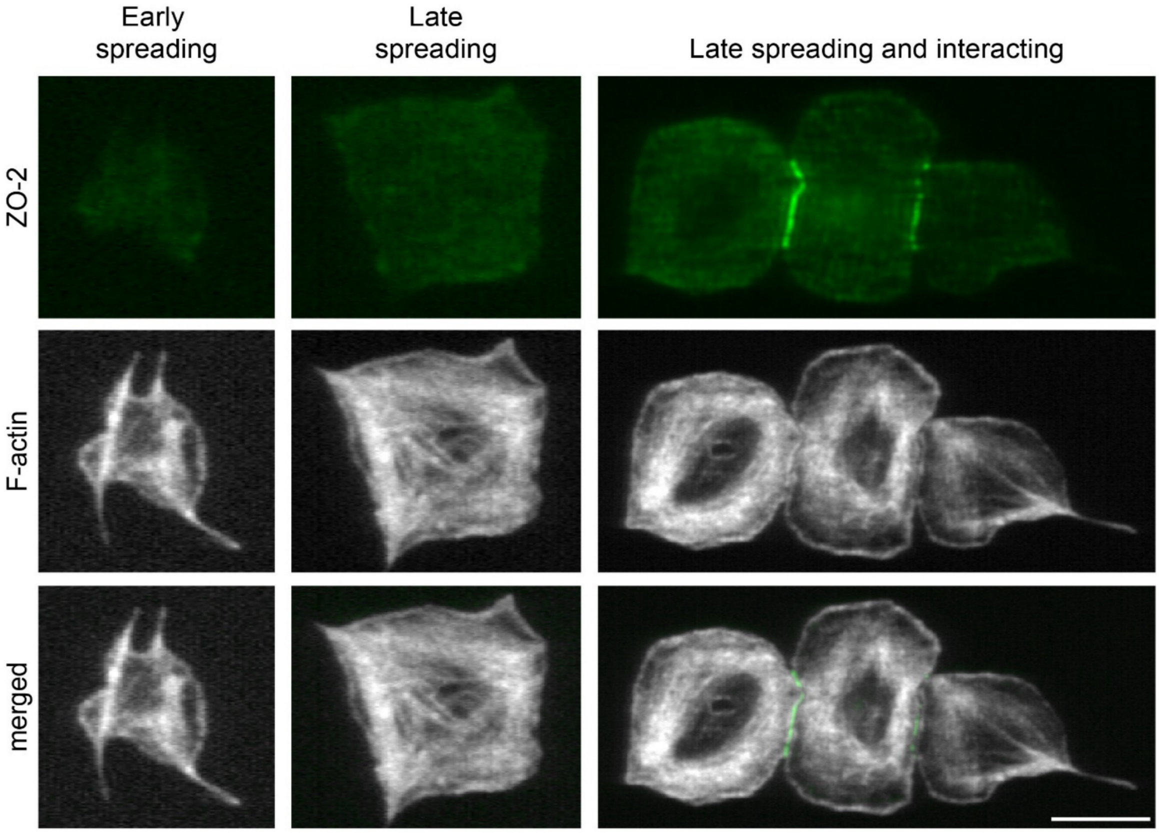


**Supplementary Figure S4. ZO-2 cluster formation primarily occurs at sites of tight platelet-platelet contact.** Representative confocal images of a ZO-2 and F-actin staining of platelets in the early stage of spreading, late stage of spreading and late stage of spreading and interaction. Images were taken with a Zeiss LSM7 system with a 100x oil immersion objective. Bar = 5 µm.

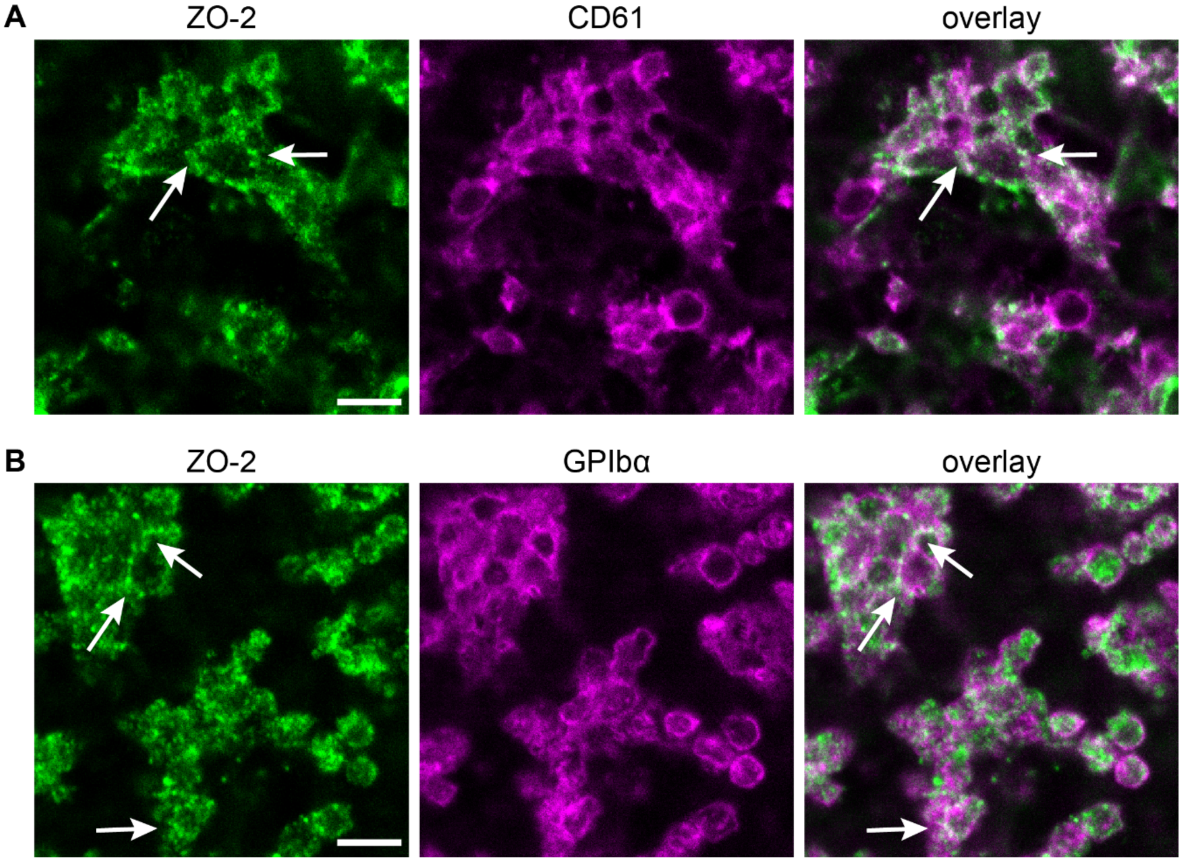


**Supplementary Figure S5. ZO-2 clustering also takes place within platelet microaggregates.** Washed platelets (500 x $10^9$/L) were allowed to spread on a fibrinogen surface in the presence of 2 mM $CaCl_2$ with additional stimulation by 1 μM 2MeS-ADP. After one hour, platelets were fixed and either stained for ZO-2 (green) and CD61 (magenta) (**A**) or ZO-2 and GPIbα (magenta) (**B**). Shown are representative confocal images of microaggregates formed on top of a monolayer of spread platelets with arrow indicating ZO-2 clusters. Images are taken at the level of the microaggregates and representative of observations made in samples from three independent platelet donors. Scale bar is 10 μm.

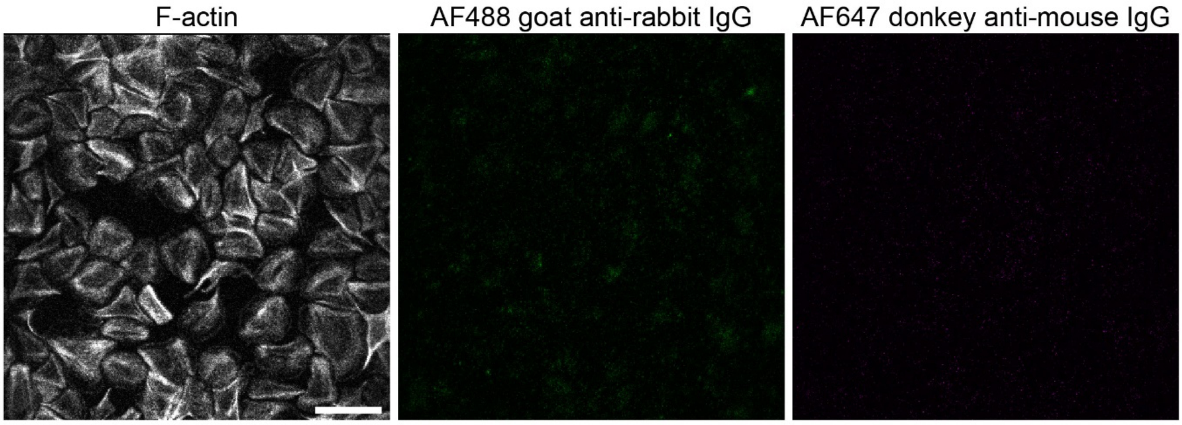


**Supplementary Figure S6. Secondary antibody controls do not show appreciable background staining.** Representative confocal images of spread platelets on a fibrinogen surface after one hour stimulation with 1 μM 2MeS-ADP (as in Figure 2, Suppl. Figure 1) stained with phalloidin (F-actin) and secondary antibodies in the absence of primary antibodies. Scale bar is 10 μm.

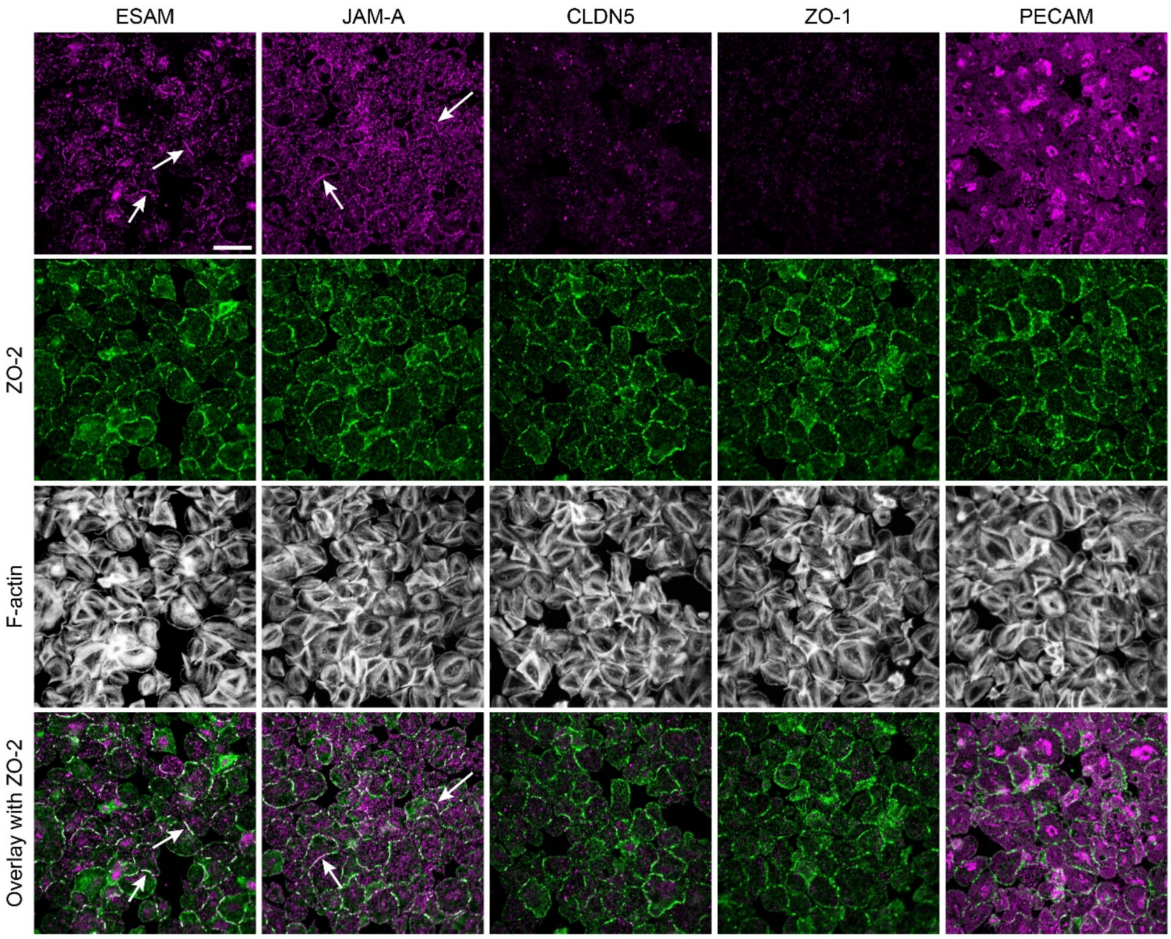


**Supplementary Figure S7. At the site of tight platelet-platelet contacts, ZO-2 colocalizes with ESAM and JAM-A.** Washed platelets (100 x $10^9$/L) spread on a fibrinogen surface in the presence of 2 mM $CaCl_2$ and 1 μM 2MeS-ADP, were fixed after one hour and prepared for immunofluorescence staining. Samples were stained for ZO-2 localization (green) and F-actin (phalloidin, gray) in combination with an ESAM, JAM-A, claudin-5 (CLDN5), ZO-1 or PECAM-1 antibody staining (magenta). Shown are representative images in full (magnifications of selected areas are illustrated in Figure 3). Scale bar is 10 μm. Arrows indicate colocalization of ZO-2 with ESAM and JAM-A.

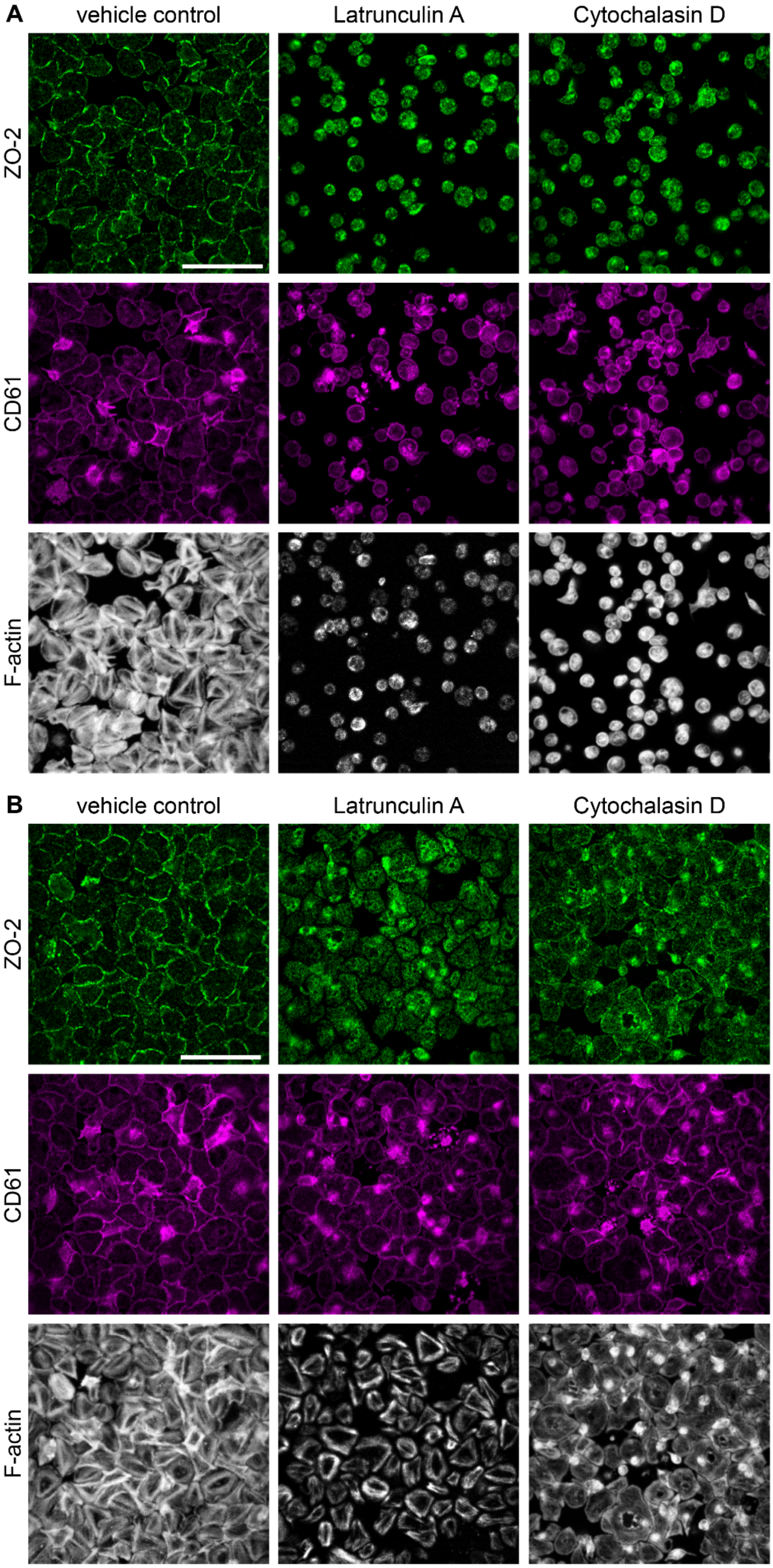
A
vehicle control
Latrunculin A
Cytochalasin D
ZO-2
CD61
F-actin
B
vehicle control
Latrunculin A
Cytochalasin D
ZO-2
CD61
F-actin

**Supplementary Figure S8. Polymerization and depolymerization of the actin cytoskeleton regulate ZO-2 clusters formation.** Washed platelets were pretreated with latrunculin A or cytochalasin D and then allowed to adhere to a fibrinogen surface in the presence of 2 mM $CaCl_2$ and 1 μM 2MeS-ADP (**A**). Alternatively, ADP-stimulated spread platelets on a fibrinogen surface were post-treated with latrunculin A or cytochalasin D (**B**). Shown are representative confocal images in full of ZO-2 staining in green, CD61 in magenta and F-actin (phalloidin) in grey. Magnifications of selected areas and quantification of ZO-2 clusters are illustrated in Figure 4. Scale bar is 10 μm.

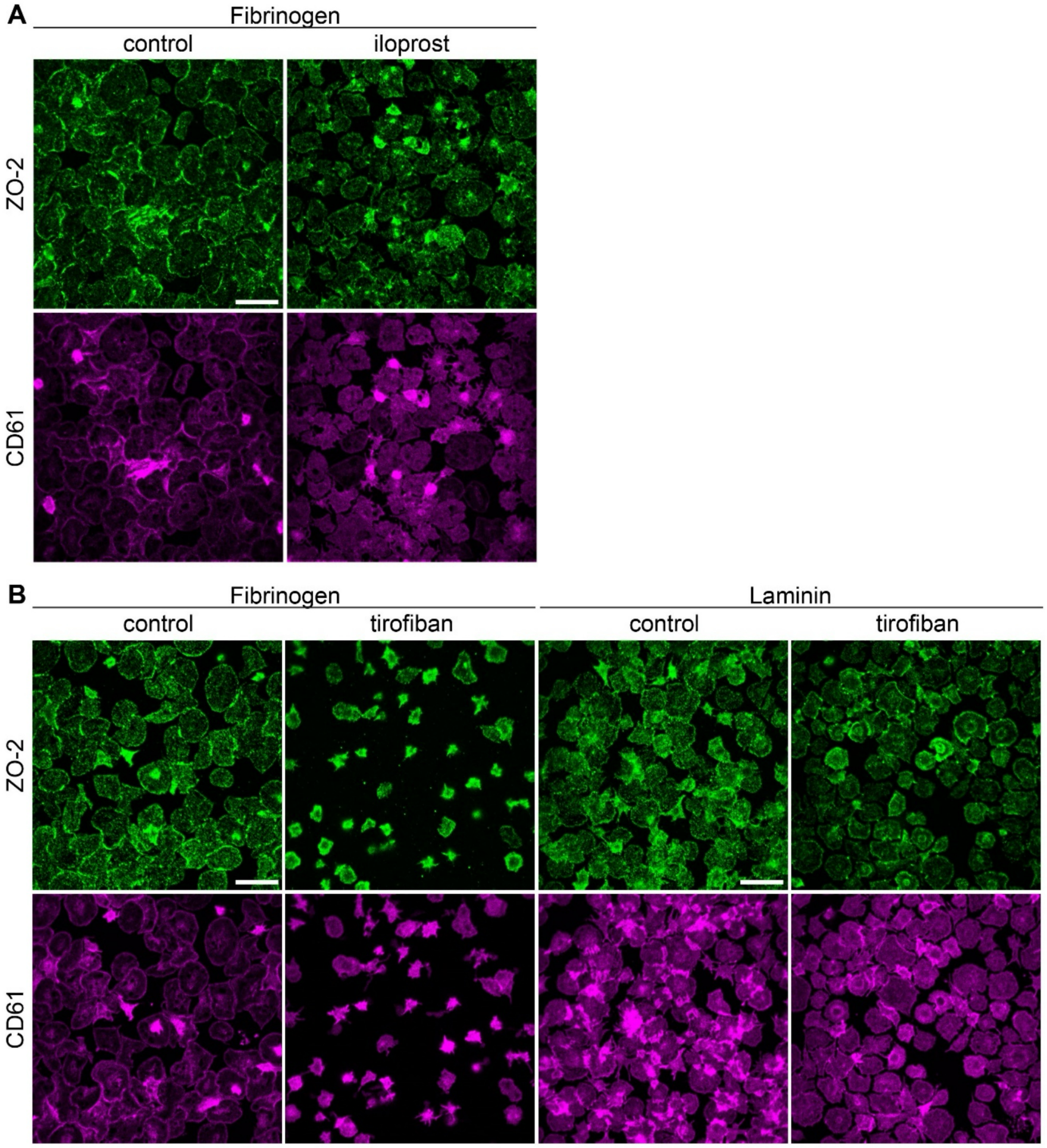


**Supplementary Figure S9. Regulation of ZO-2 clusters by cAMP and integrin $\alpha_{IIb}\beta_3$. A)** Representative confocal images of ADP-stimulated spread platelets on a fibrinogen surface post-treated with iloprost. **B)** Representative confocal images of ADP-stimulated spread platelets on a fibrinogen or laminin surface in the presence and absence of tirofiban. All samples (**A-B**) were stained for ZO-2 (green) and CD61 (magenta) localization. Magnifications of selected areas and quantification of ZO-2 clusters are illustrated in Figure 5. Scale bar is 10 µm.

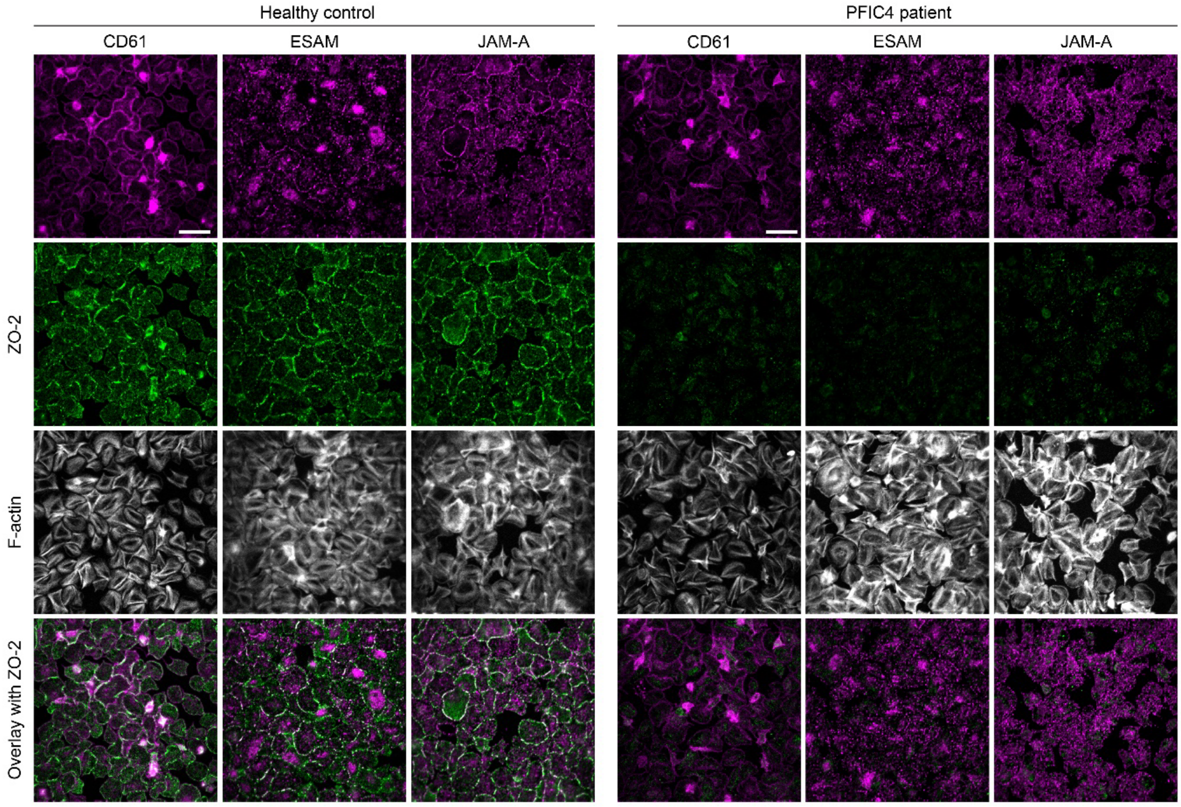


**Supplementary Figure S10. In platelets from a PFIC4 patient deficient in ZO-2, ESAM and JAM-A localization within the platelets is more diffuse.** Washed platelets (100 x $10^9$/L) from a healthy donor and a patient with PFIC4 caused by a mutation in the TJP2 gene coding for ZO-2 were spread on a fibrinogen surface in the presence of 2 mM $CaCl_2$ and 1 µM 2MeS-ADP. After one hour, platelets were fixed and stained for ZO-2 localization (green) and F-actin (phalloidin, gray) in combination with a CD61, ESAM or JAM-A antibody staining (magenta). Shown are representative images in full (magnifications of selected areas are illustrated in Figure 6). Scale bar is 10 µm.
